# SIGNIFICANT PUBLIC POLICY ISSUES IN THE HEALTH SCIENCES

**DOI:** 10.1093/geroni/igz038.1457

**Published:** 2019-11-08

**Authors:** Stephen Kritchevsky

**Affiliations:** Wake Forest School of Medicine, Winston-Salem, North Carolina, United States

## Abstract

This presentation will cover public policy issues of significance to the aging population, focusing on the perspective of the health sciences and on policies that may improve physical and mental health.

